# Logging identification and prediction of diagenetic facies in the first member of Dainan formation, Southern Gaoyou Sag, Subei Basin, China

**DOI:** 10.1038/s41598-026-35613-3

**Published:** 2026-01-09

**Authors:** Yuezhe Li, Bing Liang, Lianjun Xia, Zhenqi Wang, Juan Zhang, Lixin Fan, Ziyue Li

**Affiliations:** 1 School of Geosciences, Yangtze University, Wuhan, 430100 China; 2 China Petroleum and Chemical Corporation Jiangsu Oilfield Branch, Nanjing, 210046 China; 3 College of Geophysics and Petroleum Resources, Yangtze University, Wuhan, 430100 China

**Keywords:** Diagenetic facies, Reservoir characteristics, Logging identification, Dainan formation, Gaoyou sag, Energy science and technology, Solid Earth sciences

## Abstract

Focusing on the reservoir in the 2nd sub-member of the first member of Dainan Formation (E_2_d_1_^2^) in the southern Gaoyou Sag, Subei Basin, this study systematically analyzed the E_2_d_1_^2^ reservoir characteristics and diagenetic features on the basis of thin-section identification, scanning electron microscopy (SEM), X-ray diffraction (XRD), and petrophysical property data, and integrated analysis with well logging data was employed to investigate diagenetic facies characteristics. Results reveal that the reservoir predominantly comprises feldspathic lithic quartz sandstone. Besides residual intergranular pores, pore types include feldspar intragranular dissolution pores, lithic intergranular dissolution pores, and minor micropores (microfractures and moldic pores), and exhibit a pore-throat combination pattern characterized by the co-development of submicron- to micron-sized pores and submicron-sized throats. Combining core analysis data with diagenesis characteristics, the diagenetic facies in the study area were classified into four types: weakly compacted–weakly clay cemented dissolution facies, intensely compacted–dissolution diagenetic facies, moderately compacted–carbonate cemented diagenetic facies, and intensely compacted–clay mineral cemented diagenetic facies. Logging response templates were established to enable vertical prediction of diagenetic facies in uncored intervals. By integrating seismic data, the planar distribution of diagenetic facies was predicted, revealing that high-quality reservoirs (Weakly Compacted–Weakly Clay Cemented Dissolution Facies) are primarily distributed in the eastern and southern fan-delta front, while low-quality facies prevail in the central and western areas. This study provides a practical workflow for diagenetic facies prediction and highlights promising targets for future exploration. The study results have certain guiding significance for future exploration and development.

**Supplementary Information:**

The online version contains supplementary material available at 10.1038/s41598-026-35613-3.

## Introduction

Diagenetic facies constitute a comprehensive characterization of rock assemblages formed under specific diagenetic environments and their corresponding diagenetic attributes, serving as a critical factor in determining reservoir quality, studies on diagenesis and diagenetic facies typically employs analytical techniques such as thin-section identification, SEM, fluid inclusion analysis, and XRD, integrated with organic geochemical methods like vitrinite reflectance and gas chromatography–mass spectrometry (GC-MS)^1–4^. These methodologies aim to unravel diagenetic evolutionary sequences, mineral paragenetic relationships, and their impact on reservoir properties^5^. Nevertheless, oil-gas exploration in sedimentary basins is frequently constrained by discrete core data, limiting consecutive diagenetic facies prediction and reservoir evaluation.

The oil reservoirs in the Gaoyou Sag mainly developed in the 2nd sub-member of the first member of Dainan Formation (E_2_d_1_^2^), featuring excellent source rock conditions and the development of transmissive faults. Previous studies indicate that the timing of hydrocarbon charging in E_2_d_1_^2^ of the Gaoyou Sag primarily occurred during Intermediate diagenetic Stage A. By 2025, the first member of Dainan Formation in the Gaoyou Sag has cumulatively submitted geological reserves exceeding 63 million tons, among which the E_2_d_1_^2^ account for over 60%^6^. This is a major growth point in oil and gas exploration in recent years. In the southern part of the study area, during the sedimentary period of the E_2_d_1_^2^, fan delta deposits were mainly formed. The stable development of subaqueous distributary channels is considered to be a favorable area for oil and gas exploration. However, the E_2_d_1_^2^ in this study area mainly characterized by lithologic oil reservoirs with relatively poor porosity and permeability. Therefore, conducting research on the diagenetic facies of the E_2_d_1_^2^ is of great significance for the subsequent exploration and development of the reservoir^7–10^.

The response relationships between well-log curves such as gamma ray, neutron, and acoustic transit time and diagenetic processes enable the interpretation of diagenetic patterns through log responses, leveraging the vertical continuity of logs for continuous diagenetic facies identification. Existing logging identification methods mainly encompass cross-plot, radar-plot (spider plot), and mathematical analysis techniques, each exhibiting distinct strengths and limitations. Cross-plot analysis, the most conventional method, defines diagenetic facies boundaries by selecting two log parameters to establish a coordinate system and interpreting data point distributions; though operationally straightforward, its accuracy is limited by parameter quantity^11^. Radar-plot analysis differentiates diagenetic facies through patterns formed by multi-parameter data point linkages, offering superior accuracy to cross-plots but with greater interpretive complexity^12^.

This study comprehensively employs cast thin sections, SEM, nuclear magnetic resonance and high-pressure mercury intrusion (HPMI) analysis, integrated with well-log data, to investigate reservoir characteristics and diagenetic features within cored intervals. Log-response relationships for each diagenetic facies type were established, enabling diagenetic facies prediction in non-cored intervals through identification chart analysis. This approach realized vertical continuous identification of diagenetic facies in the target interval of the research area, providing essential geological foundations for subsequent reservoir prediction and optimal well placement optimization in the Gaoyou Sag.

## Geological settings

**Fig. 1:**
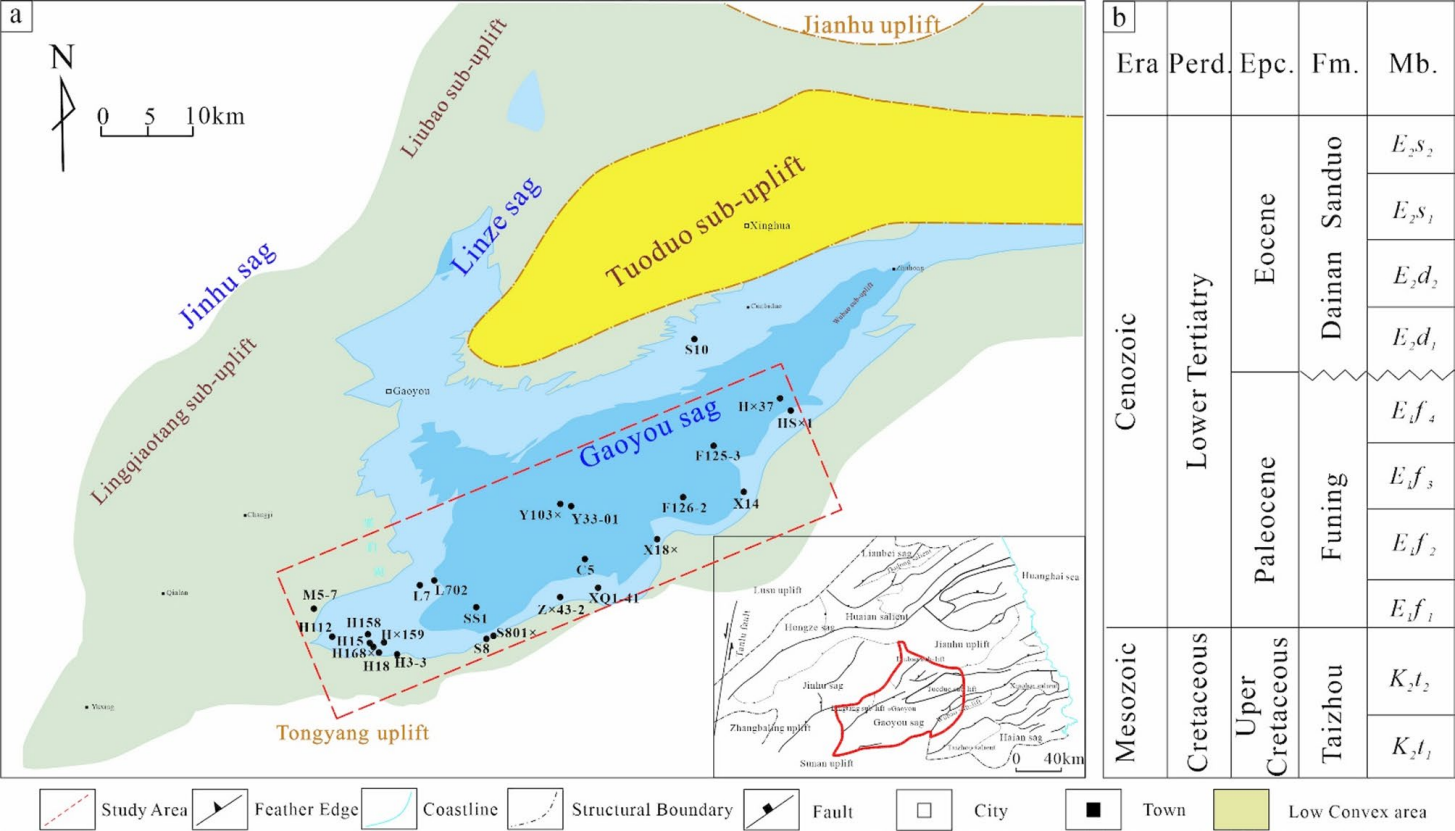
(**a**) Structure location of Gaoyou sag. (**b**) comprehensive stratigraphic column of Formation. Fm.=Formation; Mb = Member. Figure caption | This map was created by the authors based on public geographic data, using CorelDRAW Graphics Suite 2020 (Version 20.0; URL:https://www.coreldraw.com/cn/).

As the most prolific hydrocarbon-bearing sag in the Subei Basin, the Gaoyou Sag is situated in the southern part of the basin, covering an area of approximately 2,670 km²(Fig. 1a). Which is bounded by the Wubao Low Uplift to the east, the Tongyang Uplift to the south, the Linze Sag to the west, the Jianhu Uplift and Zhenduo Low Uplift to the north, and the Wubao Low Uplift to the southeast, which separates it from the Jinhu Sag. Structurally, the sag exhibits a north-south-trending configuration, comprising a fault belt, deep depression belt, and slope belt from south to north. Fault systems predominantly develop along NE, NNE, and SE orientations, collectively forming a dustpan-shaped structural style characterized by southern faulting and northern overlapping, southern deepening and northern shallowing, and southern steepness and northern gentleness^13^.

The stratigraphic sequence from the Upper Cretaceous to Quaternary includes: the Cretaceous Taizhou Formation (K₂t), Paleocene Funing Formation (E₁f), Eocene Dainan Formation (E₂d), Oligocene Sanduo Formation (E₁s), Neogene Yancheng Formation (N₂y), and Quaternary Dongtai Formation (Fig. 1b). Based on sedimentary environments and lithological associations, the Dainan Formation is subdivided into E2d1 and E2d2. The E_2_d_2_, slightly thicker than the E_2_d_1_, inherits its sedimentary framework and exhibits conformable to disconformable contact with underlying strata, with localized disconformities occurring in the northern slope. Lithologically, it consists predominantly of light gray sandstones and siltstones interbedded with brown and light gray mudstones of variable thickness, showing relatively stable lithofacies distribution^7–9^.

The Dainan Formation is overlain by the Sanduo Formation, which developed a fluvial floodplain subfacies dominated by brownish-red mudstones and grayish-white fine-to-medium-grained sandstones. The E_2_d_1_^2^ attains a total thickness exceeding 1,000 m, primarily comprising semi-deep to deep lacustrine facies. Its upper section features black mudstones intercalated with light gray sandstones, while the lower section exhibits variable-thickness interbeds of light gray sandstones and light gray to gray-black mudstones^10,13,14^.

## Samples and methods

For this study, 45 sandstone samples from the E_2_d_1_^2^ of the Dainan Formation were collected from 25 cored wells in the Gaoyou Sag. The 25 cored wells are systematically distributed across the major structural units of the Gaoyou Sag. Samples were collected from diverse structural settings, burial depths (1,600–3,500 m), and sedimentary facies, thereby ensuring the reliability and scientific validity of the dataset. After sample pretreatment and preparation, analyses including physical property tests, scanning electron microscopy (SEM), nuclear magnetic resonance (NMR), and X-ray diffraction (XRD) were conducted. Additionally, physical property data from 40 wells, 9 SEM datasets, and 14 high-pressure mercury injection datasets were obtained from the Geophysical Research Institute of Jiangsu Oilfield.

### Cast thin-section analysis

The 45 samples were made into cast thin sections, and together with 29 collected ordinary thin sections, were photographed using an RH-3230 polarizing microscope. Quantitative statistics were performed on parameters such as detrital grains, authigenic minerals, plane porosity, matrix content, grain size, and sorting.

### Physical property analysis

The 45 drilled plug samples underwent oil washing and drying to remove pore fluids. Physical property tests were then conducted using an SCMS-E high-temperature and high-pressure core multi-parameter measurement system under conditions of 28 °C and a net confining pressure of 3 MPa.

### Scanning electron microscopy (SEM)

Observations were performed using a Thermo Fisher Apreo field emission scanning electron microscope equipped with an energy dispersive spectrometer. Ten samples were prepared as fresh fracture surfaces and, after treatment, were observed under high vacuum mode (15 kV accelerating voltage, 8 mm working distance) to examine the microscopic pore structure and authigenic mineral morphology.

### X-ray diffraction (XRD)

Following the SY/T 5163 − 2010 standard, 54 samples were ground into powder (passed through a 200-mesh sieve, particle size < 75 μm) and their components were analyzed using a Thermo Fisher Apreo instrument.

### Nuclear magnetic resonance (NMR)

NMR tests were conducted on 12 oil-washed and dried plug samples using a MacroMR12-050 V NMR tight rock core analyzer. The CPMG sequence was employed with an echo time (TE) of 0.2 ms and a wait time (TW) of 6 s, number of echoes = 2048, and scans = 64. The transverse relaxation time (T₂) distribution spectrum was obtained by inverting the decay curve and used to characterize the full pore-size distribution of the reservoir.

## Results and discussion

### Reservoir characteristics

#### Petrological characteristics

**Fig. 2:**
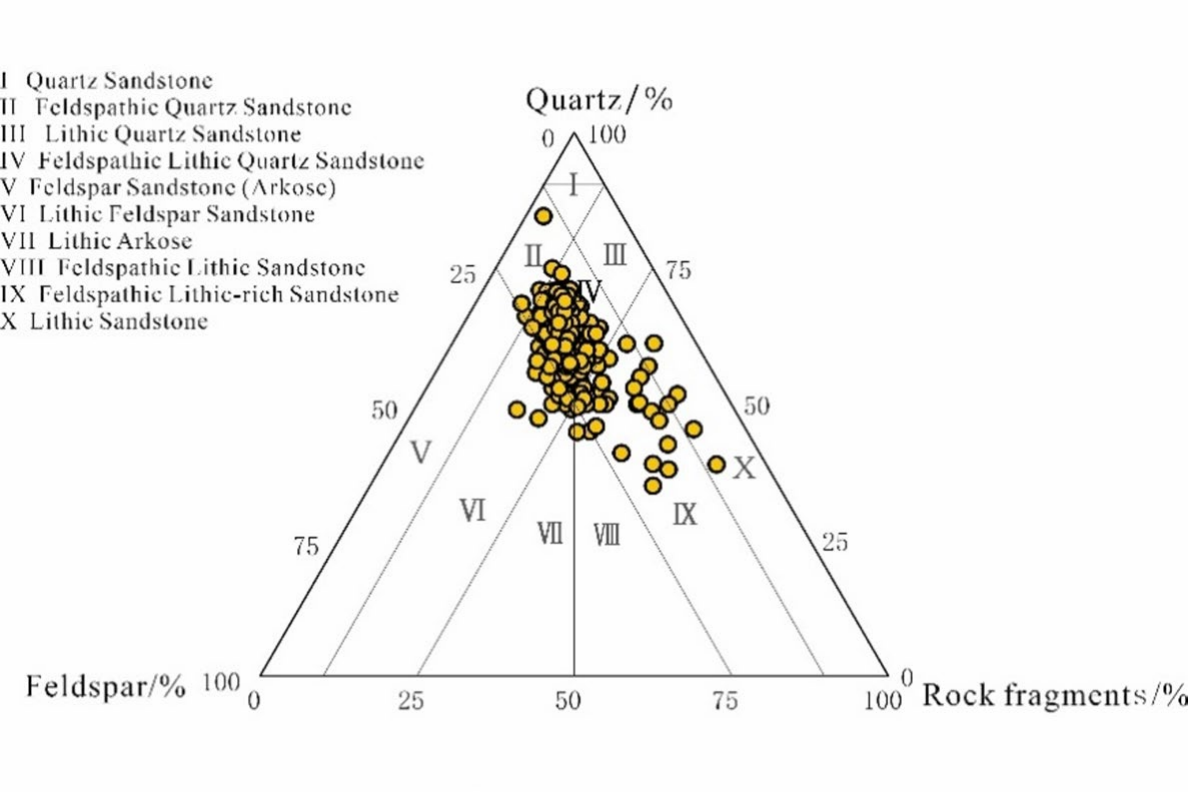
Sandstone type of reservoir of the E_2_d_1_^2^ in Gaoyou sag.

Core and cast thin section data reveal that the E_2_d_1_^2^ reservoirs in the Gaoyou Sag predominantly comprise Feldspathic Lithic Quartz Sandstone, with some Feldspathic Lithic-rich Sandstone and a small amount of Lithic Sandstone. The detrital components consist of quartz (Q), feldspar (F), and rock fragments (R) (Fig. 2). Sandstone grain composition is dominated by quartz (absolute content: 35%–84.6%, avg. 60.9%), followed by feldspar (6.7%–34.6%, avg. 19.64%) and rock fragments (2.8%–53.2%, avg. 19.46%). The argillaceous matrix content ranges from 0.2% to 25% (avg. 2.88 vol%), with a Q/(F + R) ratio spanning 0.54–5.49 (avg. 1.63). Grain roundness is predominantly subangular to subrounded, with subordinate subrounded to subangular and subangular grains, indicating high compositional maturity but moderate textural maturity. Cements are primarily contact-type, supplemented by porous and contact-porous types, while grain contacts are mainly point or point-line configurations.

**Fig. 3:**
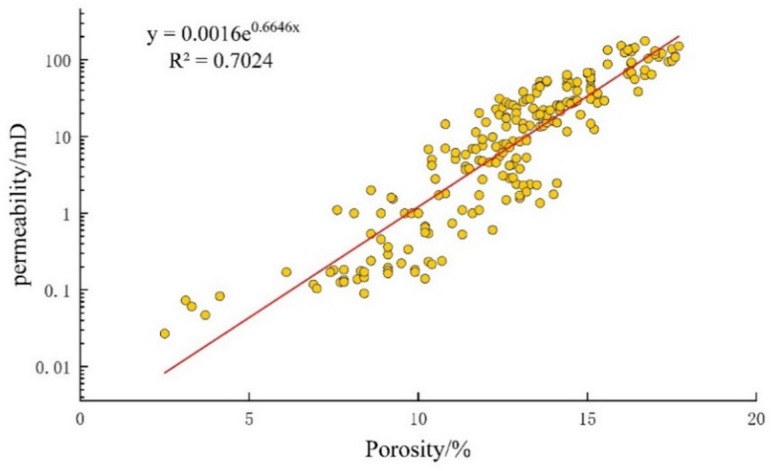
Physical properties of reservoir of the E_2_d_1_^2^ in Gaoyou sag.

There is a good linear relationship between porosity and permeability in the E_2_d_1_^2^ of the Gaoyou Depression reservoir (Fig. 3), Porosity ranges from 1.1% to 39.5% (avg. 13.4%; median 13.9%), and permeability varies between 0.007 mD and 580 mD (avg. 34.59 mD; median 12.35 mD), which has been confirmed that it is low-porosity and low-permeability reservoir. The general porosity-permeability trend indicates that pore space provides the primary pathway for fluid movement. However, the significant scatter in the data, particularly the wide permeability variation at similar porosity values, suggests that pore geometry—specifically, throat size and connectivity—exerts a secondary but critical control. In these tight sandstones, narrow or poorly connected throats likely restrict flow, leading to lower-than-expected permeability even when porosity appears moderate.

#### Reservoir space types and pore structure

**Fig. 4:**
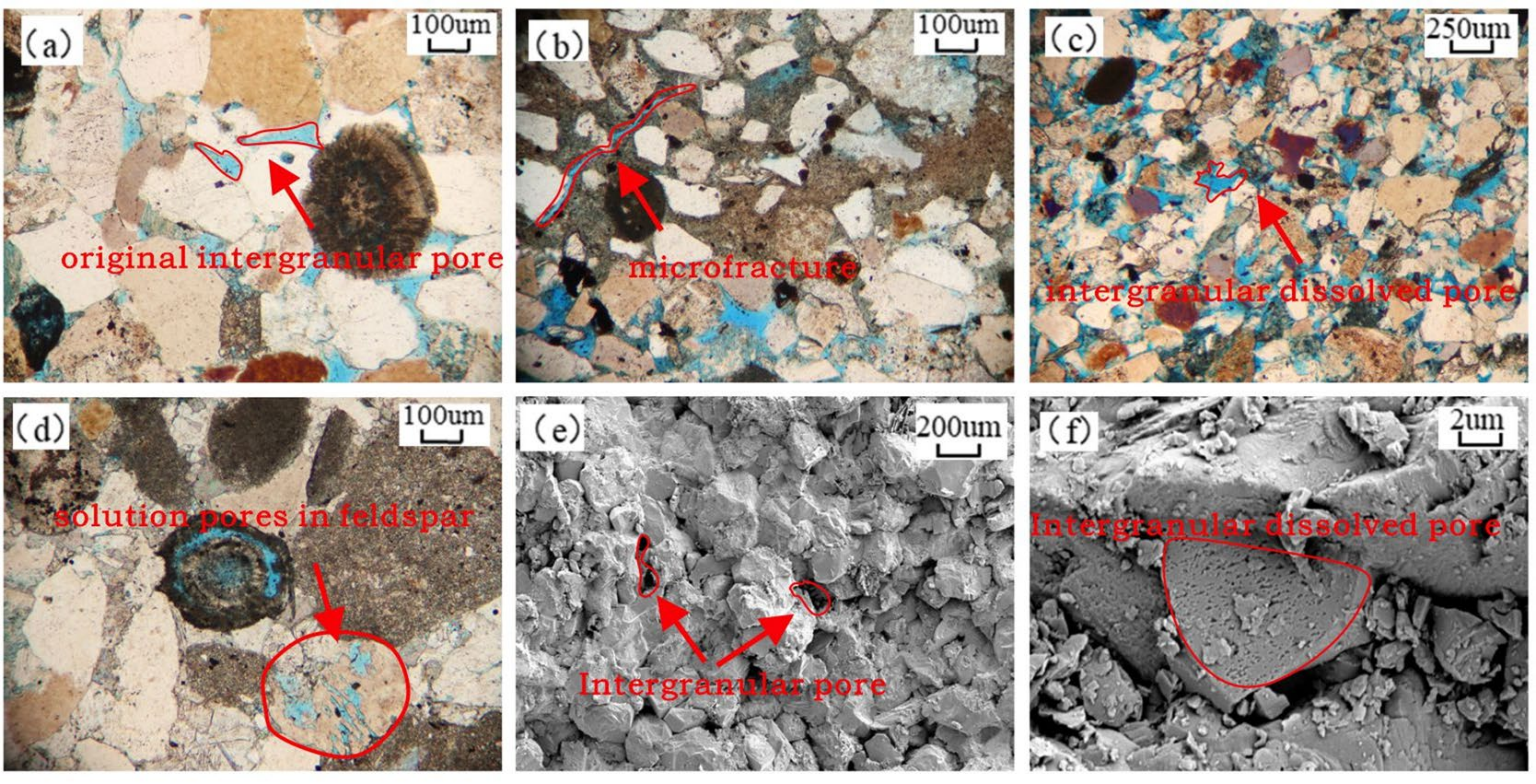
(**a**) Primary intergranular pores, Well F125-3, depth 3582.7 m, cast thin section, orthogonal light.(**b**) Microfractures, Well M5-7, depth 1942.4 m, cast thin section, orthogonal light.(**c**) Intergranular dissolution pores, Well Hdeviated-37, depth 2978.2 m, cast thin section, orthogonal light.(**d**) Feldspar dissolution pores showing partially dissolved ooids, Well HSx1, depth 3099.5 m, cast thin section, orthogonal light.(**e**) Intergranular pores, Well F126-2, depth 3663.1 m, SEM.(**f**) Intragranular dissolution pores, Well M5-7, depth 1967.4 m, SEM.

Analysis of cast thin sections and SEM data reveals a complex pore system. Primary pores, though significantly reduced by compaction, are represented by residual intergranular pores with straight boundaries (Fig. 4a). Secondary porosity, formed by the dissolution of unstable feldspar and lithic fragments, is the most significant contributor to storage space, exhibiting features, such as honeycomb-like intragranular dissolution pores in feldspar (Fig. 4d) and intergranular dissolution pores (Fig. 4c), with minor occurrences of microfractures (Fig. 4b), moldic pores, and other specialized pore types. Fractures in the study area primarily formed by tectonic activity are commonly associated with faults and predominantly distributed near fault zones^10^. Under the microscope observation, most of the cracks are in an open state, and local cracks are filled with carbonate cement formed in the late stage. Residual intergranular pores are pores that remain after compaction and cementation, and their boundaries are mostly straight and regular, significantly different from secondary pores that are harbor shaped or irregular in shape. These types of pores are relatively less developed in the study area.

The pore types in the study area result from the dual modification of the original sediments by both constructive and destructive diagenetic processes. Dissolution stands as the central constructive mechanism, playing a key role in enhancing reservoir quality. In contrast, compaction, cementation, and fracture infilling represent the principal destructive factors that degrade reservoir properties.

**Fig. 5:**
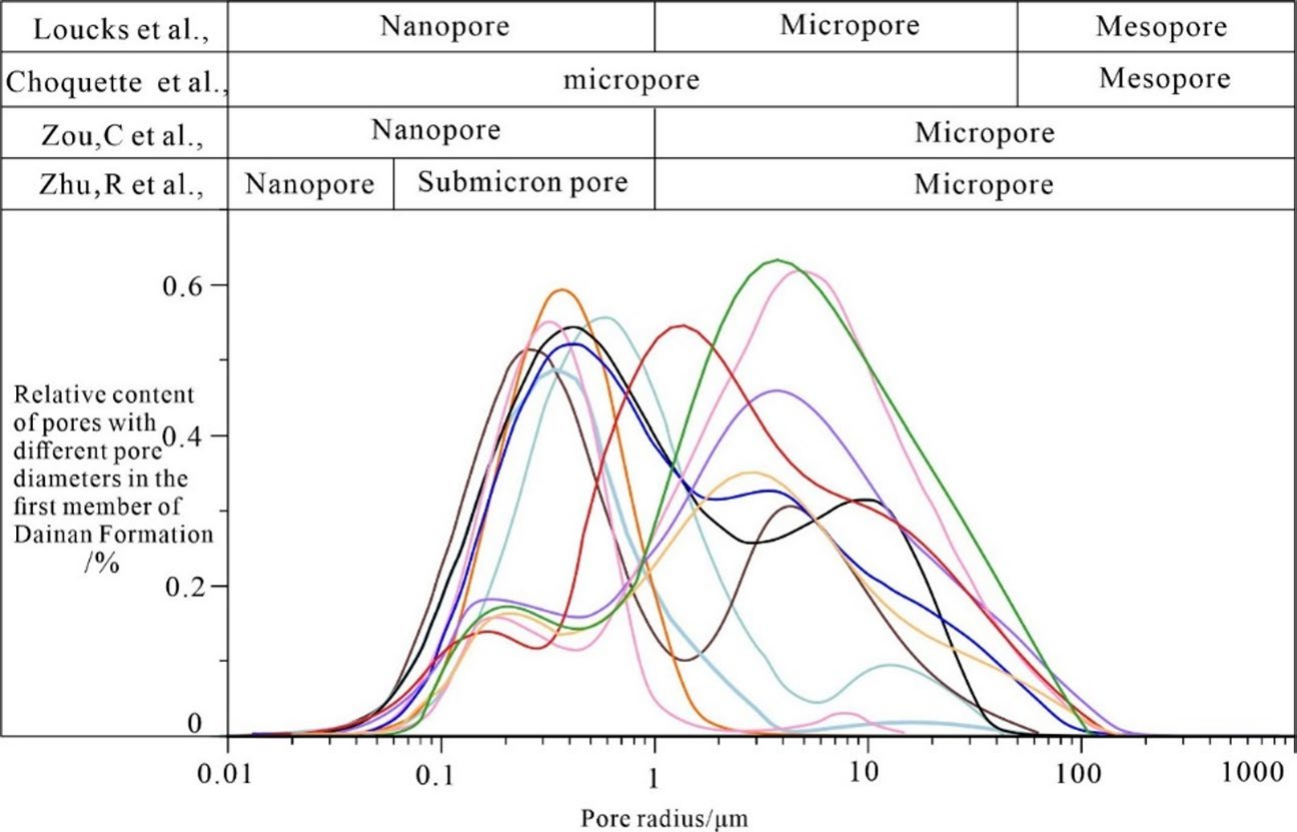
Pore size distribution of reservoir of the E_2_d_1_^2^ in Gaoyou sag.

Analysis of NMR relaxation signals reveals a prevalent bimodal distribution across the 12 samples (Fig. 5), corresponding to the pore-throat duality inherent to the reservoir’s microscopic pore architecture. Successful quantitative transformation of T₂ spectrum relaxation signals into pore-throat size distributions was achieved through joint calibration with high-pressure mercury intrusion (HPMI) cumulative porosity parameters^9,15–17^. Results demonstrate significant heterogeneity in pore structure parameters, which pore radii primarily range from 0.79 to 85 μm (mean 46.54 μm) while throat radii concentrate within 0.11–1.42 μm (mean 0.46 μm), collectively exhibiting a synergistic configuration of submicron-to-micron scale pores and submicron-scale throats. Overall pore-throat sorting and connectivity are relatively poor, evidenced by maximum mercury saturation values between 40.52% and 97.6% (mean 80.62%), displacement pressures ranging from 0.03 to 41.25 MPa (mean 19.13 MPa), pore-throat sorting coefficients varying from 0.01 to 2.89, and structure coefficients spanning 0.01 to 13.66, with respective mean values of 1.31 and 6.85.

### Diagenesis and diagenetic facies characteristics

The reservoir quality revealed by the petrological and petrophysical properties is predominantly a product of various diagenetic processes that have altered the original sedimentary fabric^18–20^. The interplay and relative timing of these processes ultimately control the pore-throat structure, giving rise to distinct diagenetic facies that map directly to reservoir quality^20–23^.

The E_2_d_1_^2^ reservoirs in the study area, predominantly buried at depths of 2,500–3,500 m, have been modified by a sequence of diagenetic events dominated by compaction, dissolution, and cementation.

**Fig. 6:**
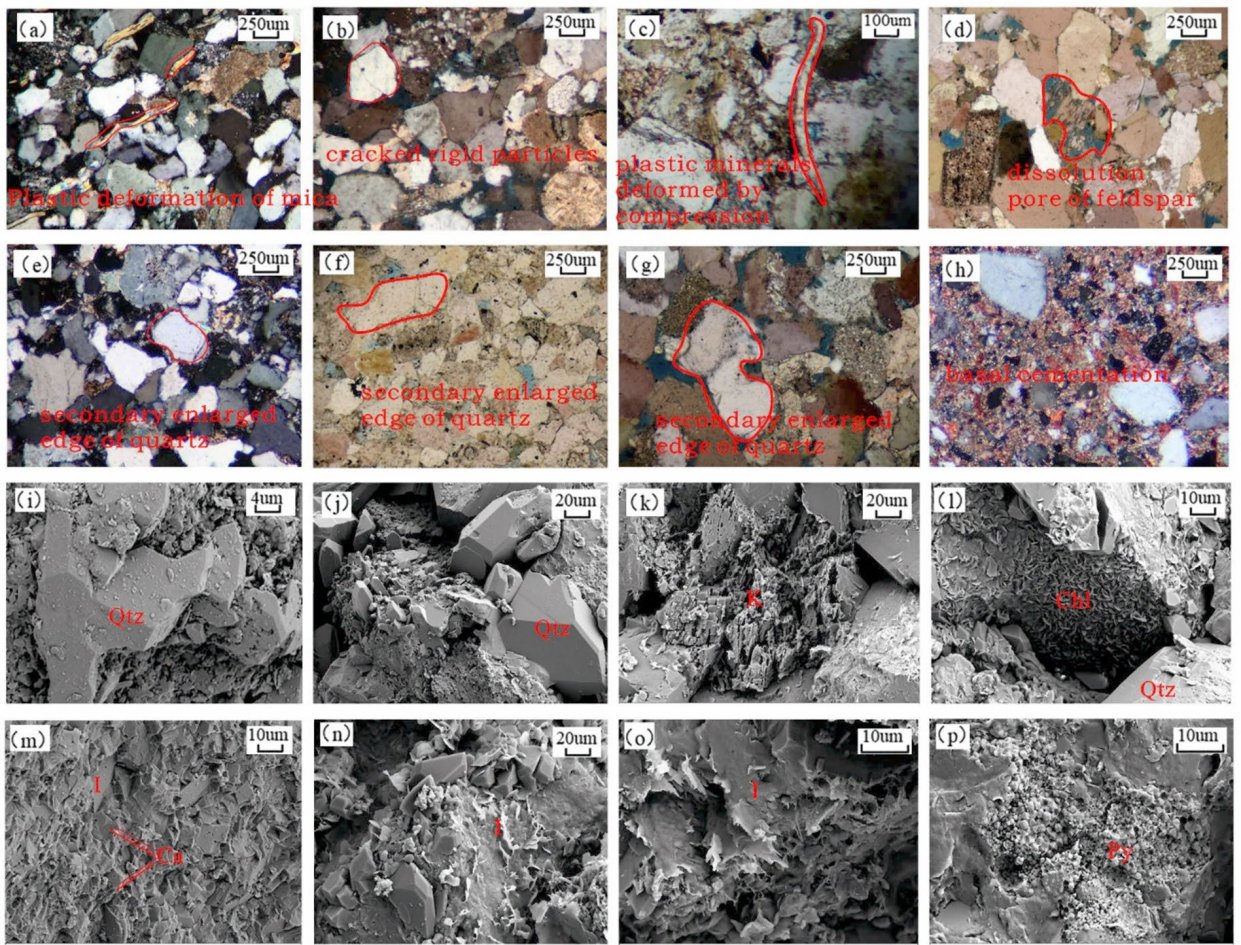
(**a**) Mica plastic deformation, Well L7, 3582.7 m, cast thin section, orthogonal light; (**b**) Rigid grain fracturing, Well M5-7, 1967.4 m, cast thin section, orthogonal light; (**c**) Mica plastic deformation, Well H15, 2209.87 m, cast thin section, plane-polarized light; (**d**) Feldspar dissolution pores, Well F126-2, 3663.1 m, cast thin section, orthogonal light; (**e**) Quartz secondary overgrowth, Well X102x, 3731.14 m, standard thin section, orthogonal light; (**f**) Quartz overgrowth cementation, Well X18x, 3535.0 m, cast thin section, plane-polarized light; (**g**) Quartz secondary overgrowth (Stage II dominant), Well F126-2, 3663.1 m, cast thin section, orthogonal light; (**h**) Basal cementation, Well H158, 3028.0 m, standard thin section, orthogonal light; (**i**) Quartz secondary overgrowth, Well M5-7, 1967.4 m, SEM; (**j**) Quartz overgrowth cementation (Stage II dominant), Well Y103x, 3561.7 m, SEM; (**k**) Feldspar leaching dissolution, Well X14, 3134.0 m, SEM; (**l**) Grain-coating leafy chlorite with quartz overgrowth, Well X18x, 3535.0 m, SEM; (**m**) Calcite-dominated cements with illite-smectite mixed layers and subordinate illite, Well S801x, 3839.8 m, SEM; (**n**) Curved and flaky mixed layer of montmorillonite, illite, and enlarged quartz on the grain surface, Well X14, 3134 m, SEM; (**o**) Intergranular/pore-lining curved illite-smectite mixed layers and filamentous illite, Well SS1, 3322.85 m, SEM; (**p**) Pyrite microcrystals and corresponding moldic pores, Well S8, 3037.0 m, SEM.

Compaction is the primary destroyer of primary porosity. Microstructural analysis reveals that detrital mineral grain contacts are dominated by line or point-line contacts, indicating persistent vertical stress. Plastic minerals display bending-twisting deformation, while rigid grains (quartz, feldspar) undergo fracturing along cleavage planes (Fig. 6a, b,c). The grain support mechanism progressively shifts from cement-supported to grain-supported. Concurrently, the contact relationships evolve from floating, to point, and then to point-line contacts as compaction intensifies, forcing grains into tighter arrangements. The resistance to this process is fundamentally influenced by the initial sediment composition, particularly its quartz content^24^.

Dissolution, the key constructive process, primarily affects feldspar and unstable lithic fragments, creating secondary porosity with surface porosity ranging from 0.14% to 6% (avg. 3%). Thin-section observations show feldspar grains dissolved into multilevel, layered honeycomb pores and banded pores with directional extension (Fig. 6d), with locally intense dissolution forming complete moldic pores (Fig. 6p). Unstable lithic fragments develop intracrystalline fenestral pores with serrated boundaries via selective dissolution (Fig. 6k). This dissolution is driven by acidic fluids, likely organic acids released during organic matter maturation in the middle diagenetic stage A, which migrate along microfractures. The potential for dissolution is predetermined by the initial content of feldspar and unstable lithics^25^.

Cementation is dominated by siliceous and carbonate types. Siliceous cementation occurs as quartz overgrowths (Fig. 6e, f,g, i,j) and pore-filling authigenic microcrystalline quartz^26^. Carbonate cementation is significant (avg. 17.8%), with early calcite forming pervasive, pore-occluding poikilotopic cement (Fig. 6h), and later dolomite/ferroan dolomite precipitating as saddle-shaped or rhombic crystal clusters in remaining pores, typically associated with pressure solution(Fig. 6m). With increasing burial depth reaching 2800–3500 m, the content of illite rises. This mineral, occurring as filiform, hair-like, or honeycomb-shaped aggregates, fills the intergranular pores (Fig. 6l, n,o). The I/S mixed-layer (illite/smectite) formed during the early diagenetic stage and is distributed within intergranular pores, mostly filling in a flocculent manner. It breaks down primary pores into multiple heterogeneous spaces, increases the tortuosity of pore throats, and consequently worsens reservoir quality .

**Fig. 7:**
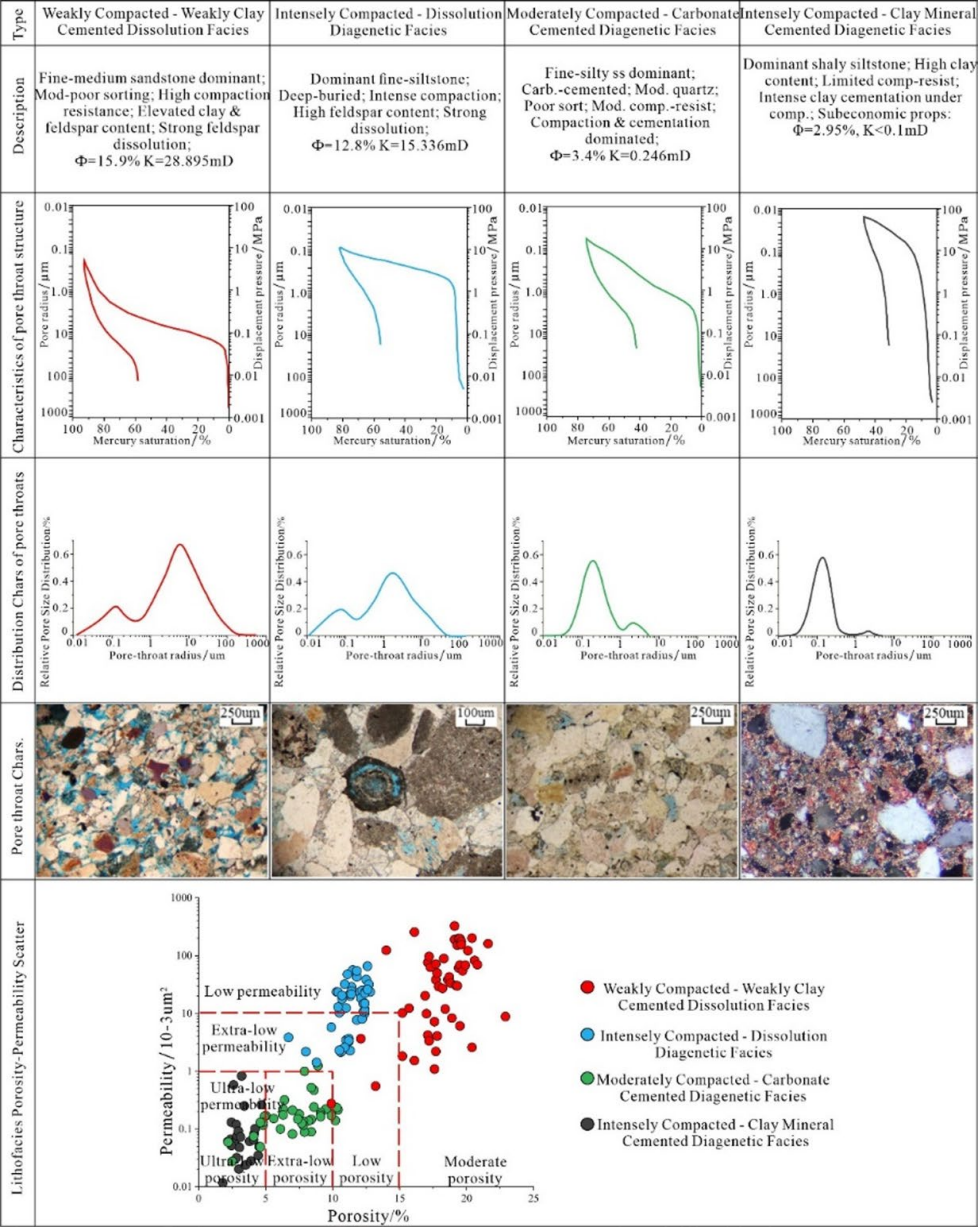
Diagenetic facies characteristic of reservoir of the E_2_d_1_^2^ in Gaoyou sag.

The type, intensity, and spatiotemporal combination of the above processes define distinct diagenetic facies, which are the genetic units controlling final reservoir quality. Based on comprehensive analysis, four dominant diagenetic facies are identified in the E2d12 reservoirs of the Gaoyou Sag (Fig. 7).

Weakly Compacted–Weakly Clay Cemented Dissolution Facies: This facies occurs in well-sorted, mid-fine sandstones of fan delta environments. Diagenesis is characterized by weak compaction, minor authigenic clay, quartz overgrowth, and significant feldspar dissolution. It exhibits the most favorable pore-throat configuration (avg. pore radius 12.64 μm) with an average porosity of 15.9% and permeability of 28.895 mD, representing the primary “sweet spot”.

Intensely Compacted–Dissolution Facies: Developing in delta-front fine sandstones, this facies underwent intense compaction followed by moderate dissolution. Pores are mainly secondary dissolution types within a compacted framework, resulting in lower average porosity (12.8%) and permeability (15.336 mD), classifying it as a low-porosity, ultralow-permeability reservoir.

Moderately Compacted–Carbonate Cemented Diagenetic Facies: Mainly distributed in nearshore subaqueous fan subfacies as fine sandstones, this facies is dominated by compaction and extensive carbonate cementation. Although cementation is strong, a high initial quartz content provides some compaction resistance. It exhibits poor pore connectivity (avg. pore radius 2.52 μm), with an average porosity of 3.4% and permeability of 0.246 mD, characterizing it as an ultra-low porosity, ultra-low permeability reservoir.

Intensely Compacted–Clay Mineral Cemented Facies: This facies develops in clay-rich, muddy silty fine sandstones of the delta front. Intense compaction is followed by cementation from illite and mixed-layer clays, which fill pore spaces. The pore-throat system exhibits extremely poor connectivity (avg. pore radius 1.1 μm), leading to an average porosity of 2.95% and permeability generally below 0.1 mD, establishing it as a non-productive, ultralow-porosity and ultralow-permeability reservoir.

The spatial distribution of these facies, governed by the interplay of original composition, burial history, and fluid flow, controls the fundamental heterogeneity of reservoir quality in the study area, with the dissolution-dominated facies defining the exploitable “sweet spots” within the system.

**Fig. 8:**
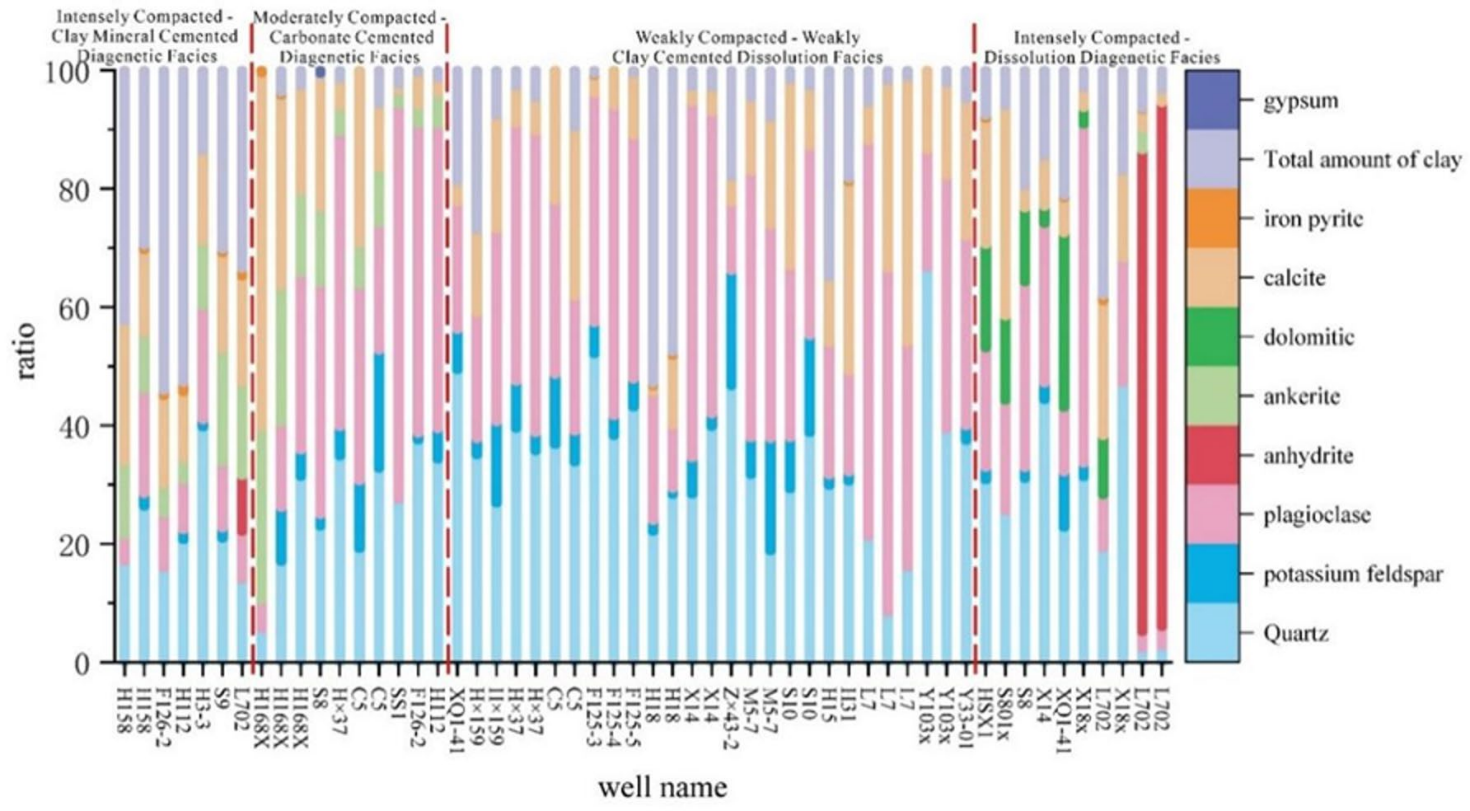
Histogram of mineral composition.

XRD analyses reveal mineralogical controls on reservoir quality^24,27^. Intensely Compacted–Clay Mineral Cemented Facies shows the highest clay content (avg.), where intense compaction destroys primary pores while clay minerals fill residual spaces, creating the poorest reservoir; Moderately Compacted–Carbonate Cemented Diagenetic Facies contains > 50% carbonate minerals that severely occlude pores, compounded by moderate compaction damage to primary porosity; Intensely Compacted–Feldspar Dissolution Diagenetic Facies, dominated by quartz with subordinate feldspar, experiences strong compaction but preserves limited primary pores due to rigid mineral content, while substantial secondary porosity develops through dissolution; Weak Compacted–Weakly Clay Cemented Dissolution Facies exhibits optimal properties due to quartz/feldspar dominance, weak compaction preserving primary pores, feldspar dissolution creating secondary porosity, and well-connected pore-throat structures, collectively forming the study area’s highest-quality reservoir (Fig. 8).

### Logging responses and prediction of diagenetic facies

#### Log response characteristics

Due to various diagenetic processes, there are significant differences in logging response characteristics between different types of diagenetic reservoirs^28–31^. The Weakly Compacted–Weakly Clay Cemented Dissolution Facies exhibits a characteristic “two-highs-one-low” signature: low natural gamma ray (GR) values averaging 60 API (range: 48–68 API) influenced by silty mudstones, siltstones, and fine sandstones; high interval transit time (AC) averaging 275 us/m (range: 240–305 us/m) attributed to well-preserved primary pores and abundant secondary dissolution pores from feldspar/lithic leaching that collectively reduce acoustic velocity^32^; and elevated Neutron(CNL) responses averaging 21% (range: 17–25%) reflecting substantial clay-bound water effects in clay-rich intervals.

The main manifestations of intensely compacted-dissolution facies are: (1) Low natural gamma ray (GR). The lithology of reservoirs with strong compaction dissolution phase is mainly fine sandstone or siltstone, with low mud content and generally low GR values. (2) Moderate interval transit time (AC). Although strong compaction destroyed some primary pores, a small amount of secondary pores were formed by later dissolution, resulting in good overall physical properties and moderate AC value. (3) Response characteristics of low CNL. This type of reservoir has a low content of clay minerals, relatively “pure” pores, and basically no influence of clay mineral bound water. Therefore, the typical logging response characteristics of strong compaction dissolution reservoirs are characterized by “two lows and one middle”.

**Fig. 9:**
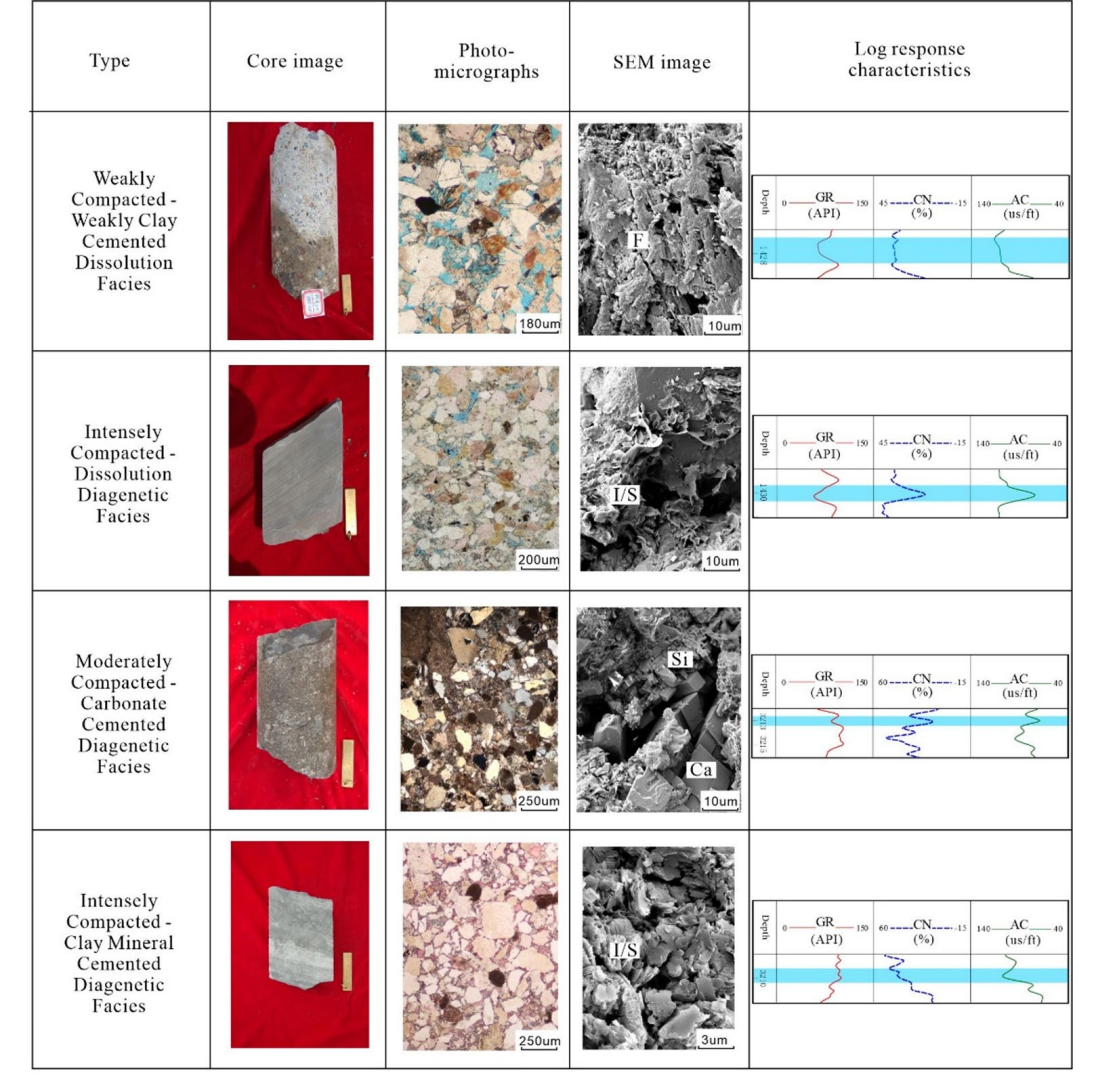
Logging response characteristics of different diagenetic facies.

The main manifestations of moderately compacted-carbonate cemented facies are: (1) Moderate GR logging response value. The lithology of this reservoir is mainly composed of quartz sandstone, with low mud content and rich carbonate cementation, which reduces the logging response value of GR. (2) Response characteristics of low CNL. This type of reservoir is mainly developed in the sedimentary subfacies of the delta front, with cement mainly consisting of contiguous calcite. Although early carbonate cement can resist certain compaction effects, the compaction degree of this type of reservoir is not high. However, in the later stage, a large amount of carbonate cement also destroys the primary pores, resulting in a deterioration of reservoir properties. (3) Low AC. The propagation speed of sound waves in carbonate minerals is much higher than that in sandstone skeletons, and the cementation of carbonate rocks destroyed the physical properties of reservoirs, weakening the ability to slow down sound waves. Therefore, when the reservoir is rich in carbonate minerals, the acoustic time difference will significantly decrease. Therefore, the typical logging response characteristics of moderately compacted-carbonate cemented facies reservoirs are characterized by “two lows and one middle”.

The main manifestations of reservoirs with strong compaction clay mineral cementation are: 1 High natural gamma ray (GR). This type of reservoir is mainly developed in low-energy mud rich environments, rich in clay minerals, and has a high GR. 2. High AC. Although this type of reservoir has a high content of plastic particles and a low content of rigid particles, and the strong compaction phenomenon of plastic particle compression can be observed under the Microscope, due to the presence of a large number of micropores and bound water in clay mineral, sound waves are reflected multiple times at the interface between clay particles and pore water, prolonging the propagation path and increasing the time difference of sound waves. 3. Response characteristics of high CNL. The hydroxyl groups and bound water in clay minerals significantly increase neutron deceleration ability, manifested as an abnormal increase in CNL. As a result, the logging response characteristics of typical intensely compacted clay mineral cemented reservoirs exhibit the “three highs” feature(Fig. 9).

#### Diagenetic facies identification and application

For wells lacking core data or laboratory analyses, vertical identification of diagenetic facies using well logs provides an effective method. Integrating core-derived data (thin sections, SEM, XRD) with log responses enables construction of identification chart for intuitive facies discrimination^28,33,34^.

**Fig. 10:**
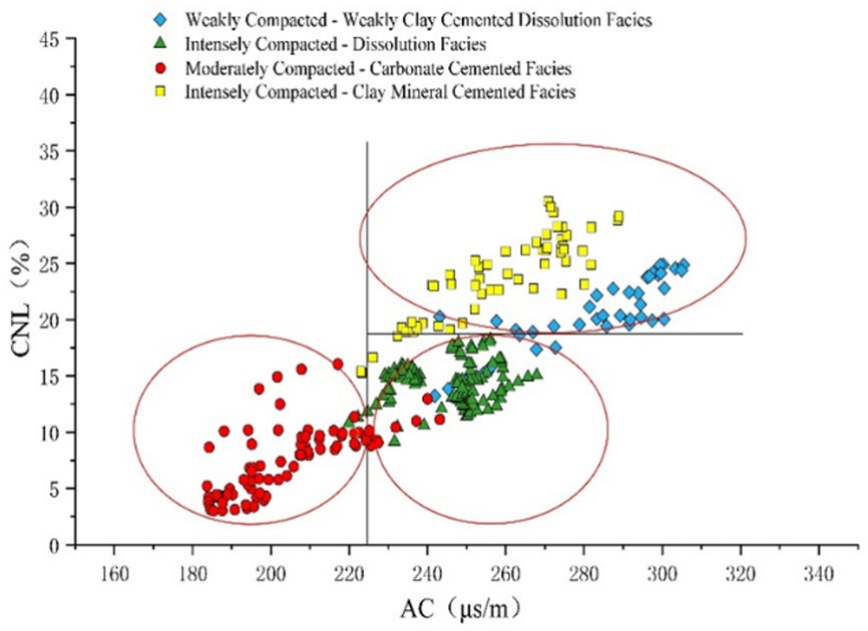
CNL-AC logging identification chart.

**Fig. 11:**
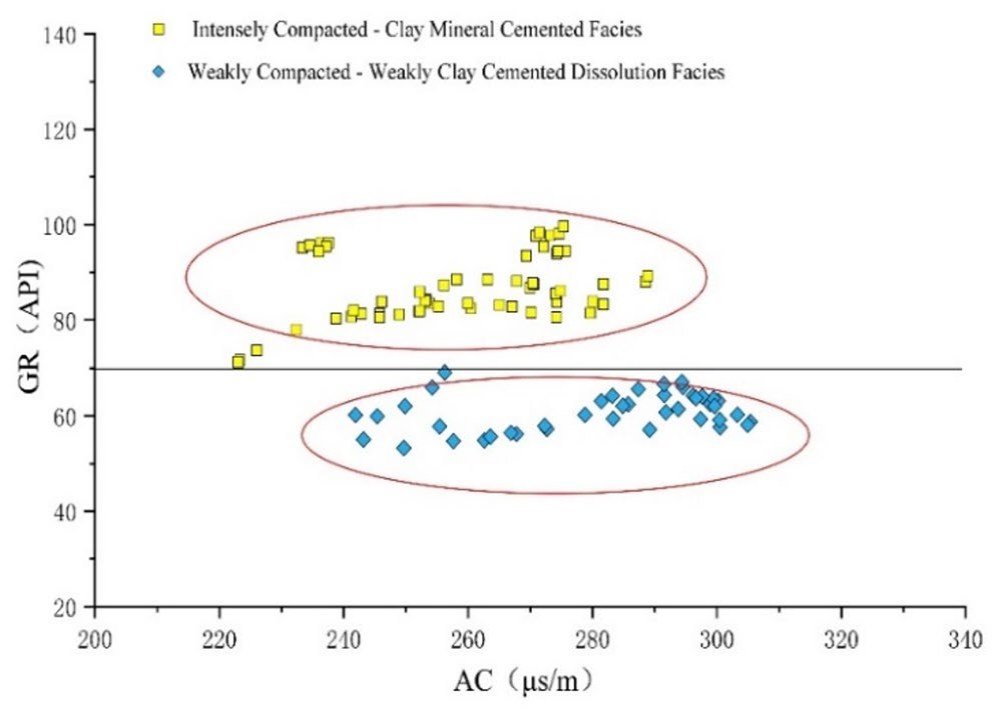
GR-AC logging identification chart.

Study reveals distinct clustering patterns for each diagenetic facies on specific log identification chart(e.g., AC-CNL, AC-GR). Samples from each facies concentrate within definable confidence intervals, creating obvious distribution trends that offer robust visual visualization basis. The combined use of AC-CNL (Fig. 10) and AC-GR (Fig. 11) identification chart effectively distinguish all four diagenetic facies across the study area.

**Fig. 12:**
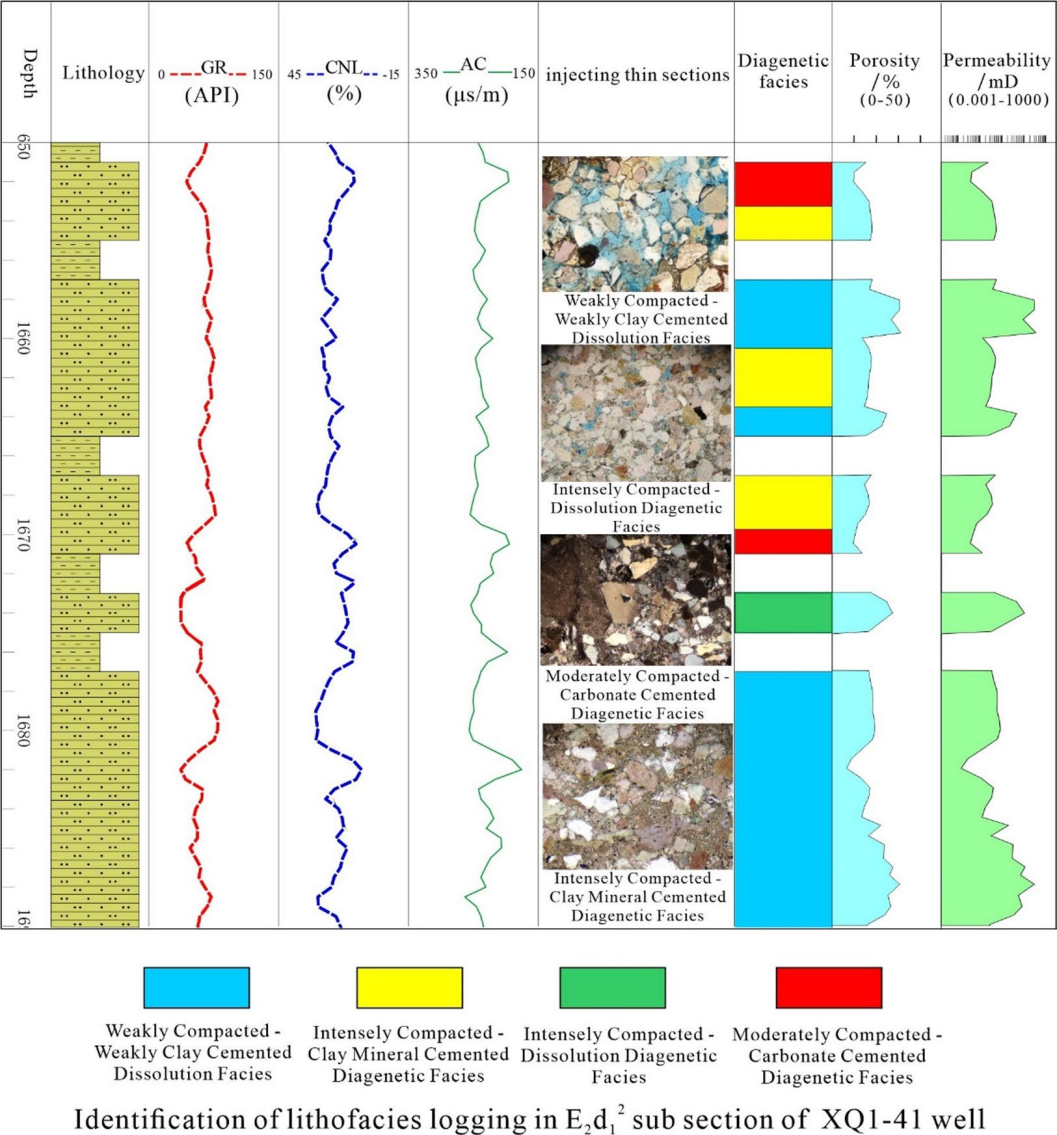
Prediction performance of Well XQ1-41.

Validation of the diagenetic facies prediction model in Well XQ1-41 demonstrates robust performance through integrated log-thin section analysis. 1657–1660 m, the diagnostic log triad of low GR, high AC, and high CNL identifies the Weakly Compacted–Weakly Clay Cementated Dissolution diagenetic Facies, which has a large number of primary intergranular pores and intergranular and intragranular dissolved pores are visible under the microscope, with good physical properties. 1660–1663 m, Presenting high GR, high AC, and high CNL logging curve characteristics, it is identified as a strong compaction clay mineral cementation phase, with plastic minerals such as mica being compressed and deformed, visible chlorite film cementation, rare pores, and basically no reservoir performance; 1673–1675 m, low GR, medium AC, and low CNL responses align with the Intensely Compacted–Dissolution Diagenetic Facies, with Quartz feldspar has a high content and strong resistance to compaction, retaining some primary pores. The dissolution of feldspar forms a small amount of secondary pores, and the reservoir has good physical properties. 1680–1687 m, Identified as a Moderately Compacted–Carbonate Cemented Diagenetic Facies, the cementation material is mainly carbonate minerals, filling intergranular pores and less developed dissolution pores, with basically no reservoir performance (Fig. 12). The results indicate that the predicted results have good consistency with the core data and experimental test results.

**Fig. 13:**
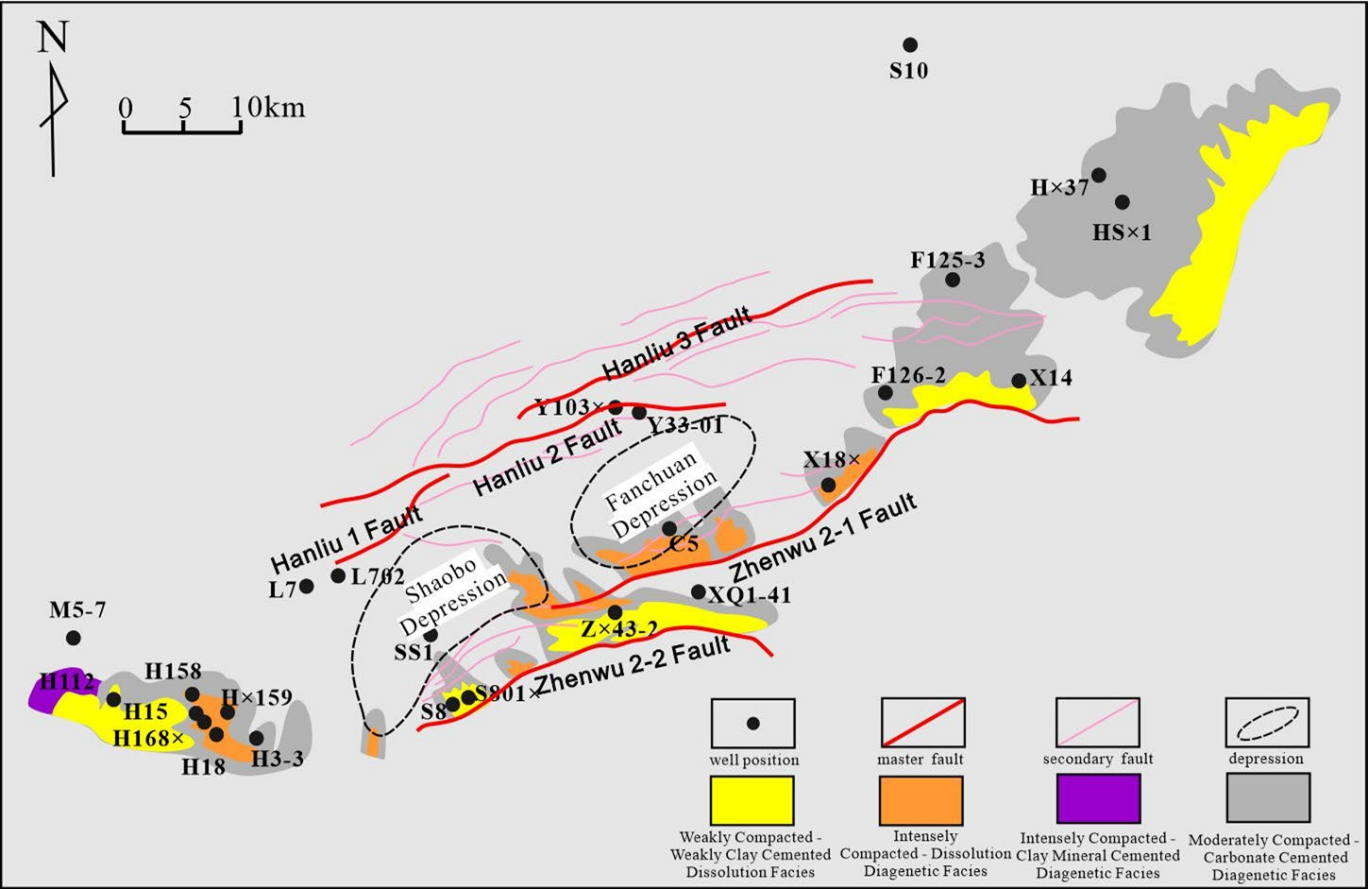
distribution of diagenetic facies.

There is a direct relationship between different diagenetic facies types and reservoir properties. Through well-to-seismic calibration, the clustering patterns of different diagenetic facies in cross-plots of seismic elastic parameters are analyzed to train a classification model. Pre-stack simultaneous inversion is then performed on 3D seismic data to generate full-field elastic parameter volumes. Finally, the trained model is applied to the inverted volumes to predict the diagenetic facies point by point, which are statistically summarized along the target horizon to produce a planar distribution map^35–39^,which is of great significance for the distribution research of sweet spot oil layers. The sandstone source in the research area mainly comes from the southern eastern part. According to X-ray diffraction, microscopic thin section and other data, the phenomenon of the secondary concrescence of quartz edge is common. The central and southern parts are mixed areas of carbonate minerals and clay minerals, with high content of illite and kaolinite. Different mineral compositions lead to significant differences in the distribution of diagenetic facies types on the plane. Sandstone with thick sand bodies distributed in the front edge of fan delta has a high quartz content and strong resistance to compaction. This type of sand body only contains a small amount of cement, and the pore type is mainly intergranular pores, with a small portion of pores filled or dissolved by clay minerals. In the plain of the fan delta, sandstone with low quartz content and thin sand body thickness reduces intergranular pores due to its high filler content. The pore types are mainly intergranular pores + intergranular pores and intergranular pores + dissolution pores. The clay mineral content in the western part of the research area is relatively high, and a large number of intergranular pores are filled with illite. Although there are some micropores and intergranular pores, the connectivity is poor and the permeability is low(Fig. 13).

#### Porosity calculation model based on diagenetic facies classification

Due to the significant differences in logging response characteristics among reservoirs of different diagenetic facies types (Fig. 13), directly establishing a porosity calculation model using core scale logging will result in poor model correlation and unsatisfactory calculation results^40–48^. Therefore, based on the classification of diagenetic reservoirs, porosity calculation models were established for four types of reservoirs in this study. The results indicate that the porosity calculation model based on diagenetic facies classification has good correlation (Table 1). The established predictive models for the four types of diagenetic facies generally exhibit positive correlations, though their prediction accuracy varies. Higher predictive precision is observed in facies dominated by dissolution, indicating the dominant role of secondary pore development. Significant data scatter is present in some models, particularly in facies dominated by compaction and cementation. This is mainly attributed to internal heterogeneity within the diagenetic facies and the limitation that facies classification alone cannot fully capture the complexity of pore-throat architecture. These models are suitable for regional trend evaluation and “sweet spot” identification, but their predictive accuracy decreases in strongly cemented or intensely compacted intervals. It is recommended to integrate them with advanced well-log data calibrated by core measurements for reliable local predictions.

**Fig. 14:**
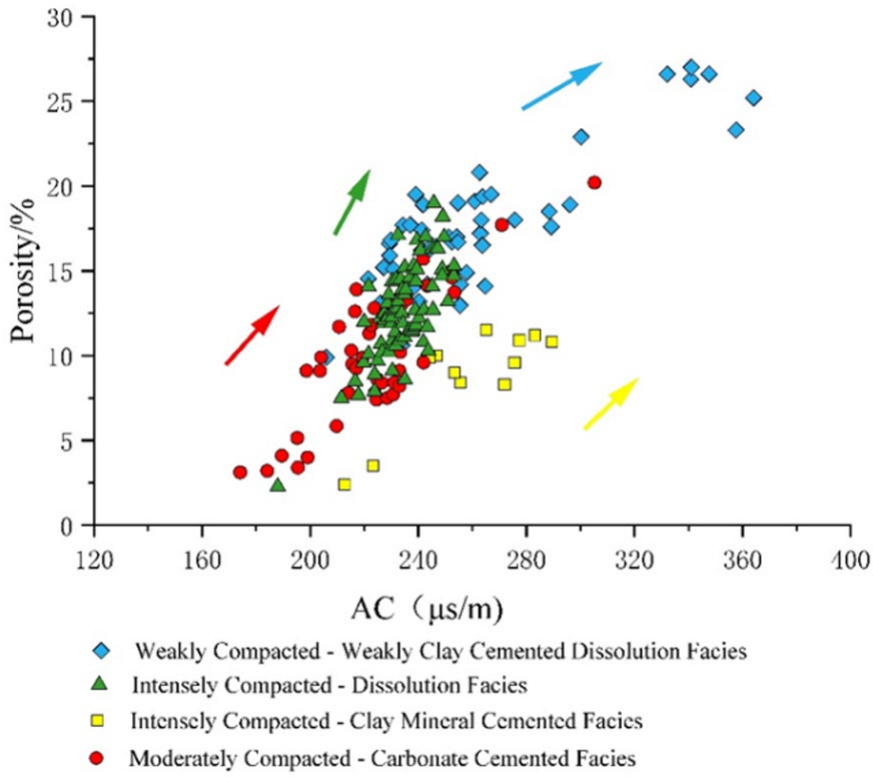
AC-porosity identification chart.

**Table 1:** Porosity computation model.

Type of diagentic facies	Porosity computation model	Correlation Coefficient
Intensely Compacted-Clay Mineral Cemented Facies	φ = 0.1031*Δt-17.839	*R* = 0.8335
Moderately Compacted-Carbonate Cemented Facies	φ = 0.10389*Δt-20.98	*R* = 0.8244
Weakly Compacted - Weakly Clay Cemented Dissolution Facies	φ = 0.0897*Δt-5.9044	*R* = 0.8503
Intensely Compacted - Dissolution Facies	φ = 0.1927*Δt-32.511	*R* = 0.7234

The porosity calculation results of the four types of models were compared and validated with the porosity analysis of the rock core (Fig. 14), further verifying the effectiveness of the porosity calculation model.

**Fig. 15:**
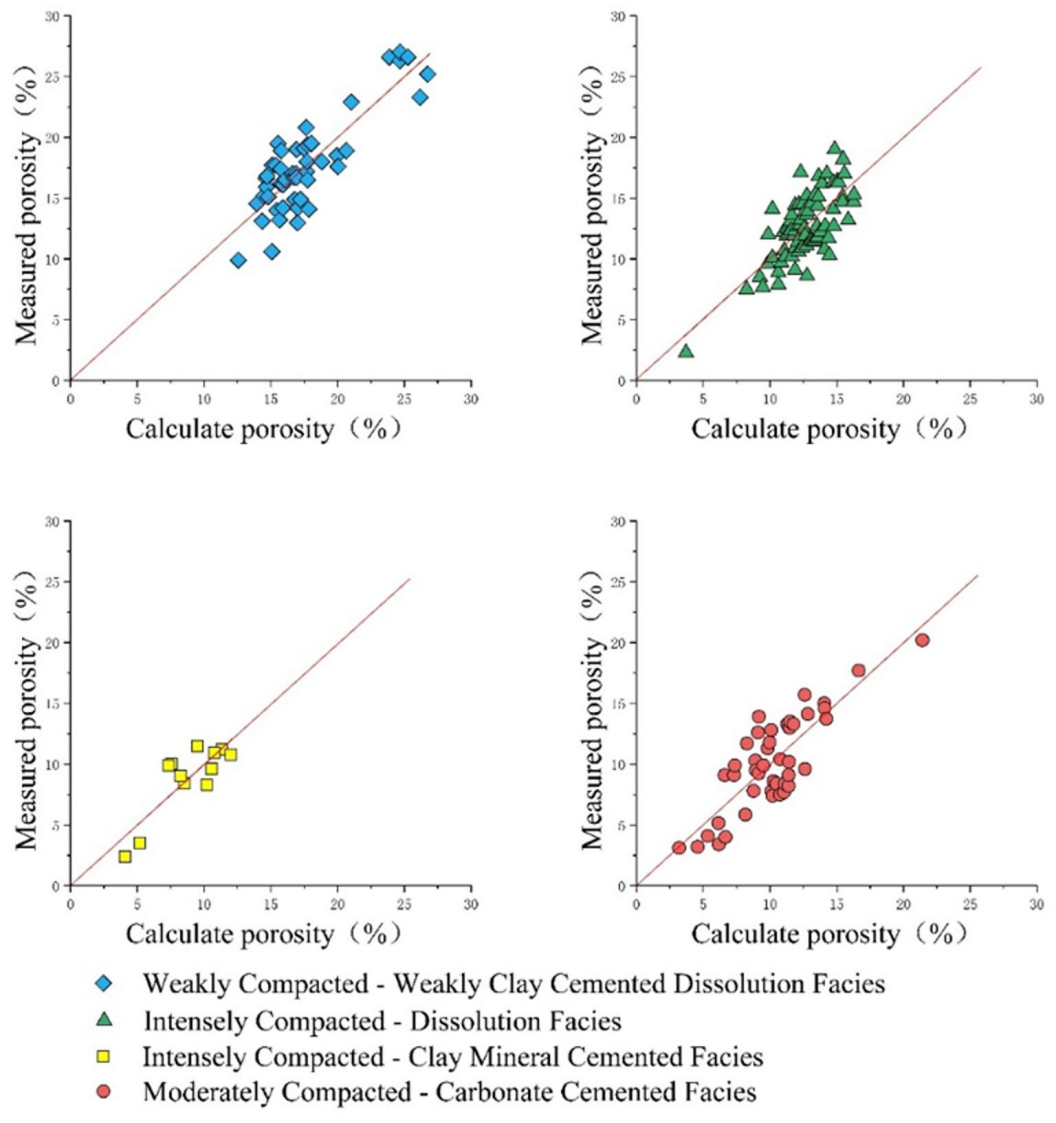
Model validation.

The validation plot (Fig. 15) demonstrates a generally good agreement between the calculated and measured porosity across all diagenetic facies. The highest correlation coefficient (R = 0.8503) is observed in the Weakly Compacted Facies, likely due to its well-connected pore system and relatively homogeneous pore structure, which is more accurately captured by acoustic log responses. Slightly lower correlations in the other facies, particularly the Intensely Compacted–Dissolution Facies (R = 0.7234), may be attributed to the greater heterogeneity in pore throat structures and the complex interplay between dissolution enhancement and compaction reduction of porosity. These facies-specific models significantly improve porosity prediction accuracy compared to a single global model, underscoring the necessity of diagenetic facies classification as a prerequisite for reservoir quantification.”

## Conclusions

(1)The E_2_d_1_^2^ reservoir of the research area is mainly composed of feldspathic lithic quartz sandstone, which exhibits the characteristics of low porosity and permeability. The storage space is mainly composed of intragranular dissolution pores, intergranular dissolution pores, and residual intergranular pores, with locally developed fractures. The intergranular micropores have a small radius and poor connectivity, making them ineffective pores. The pore radius is concentrated in the range of 0.79-85 µ m, and the throat radius is between 0.11 and 1.42 μm, showing an overall combination of micrometer sized pores and submicron sized throats.

(2)The diagenetic processes experienced by the E_2_d_1_^2^ reservoir in the research area mainly include compaction, cementation, and dissolution. According to the influence of diagenesis on reservoirs, the diagenetic facies of reservoirs can be divided into four types: Weakly Compacted–Weakly Clay Cemented Dissolution Facies, Intensely Compacted–Dissolution Facies, Moderately Compacted–Carbonate Cemented Facies, and Intensely Compacted–Clay Mineral Cemented Facies. Among them, the Weakly Compacted–Weakly Clay Cemented Dissolution Facies has the best physical properties, followed by the Intensely Compacted–Dissolution Facies and the Intensely Compacted–Clay Mineral Cemented Facies, the Moderately Compacted–Carbonate Cemented Facies have the worst physical properties.

(3)Based on the differences in logging response characteristics of different diagenetic facies reservoirs, AC-CNL and AC-GR combination identification charts were established. Four porosity calculation models were established based on the classification of diagenetic reservoirs, and the results showed that the porosity calculation models based on diagenetic facies classification had better correlation and higher calculation accuracy. Integrated seismic data analysis further enabled planar distribution prediction of diagenetic facies across the study area, revealing that favorable diagenetic facies predominantly concentrate in the eastern and southern sectors, mainly developed in the front edge of fan delta subfacies; while poor-quality facies cluster in central and western regions, mainly developed in the fan delta plain subfacies. The study results have certain guiding significance for future exploration and development.

## Supplementary Information

Below is the link to the electronic supplementary material.

Supplementary Material 1

## Data Availability

The datasets used and/or analysed during the current study available from the corresponding author on reasonable request.
